# Persistent Tachypnea and Alveolar Hemorrhage in an Infant: An Unexpected Etiology

**DOI:** 10.1155/2016/3168257

**Published:** 2016-11-08

**Authors:** John Bishara, Angela Webb, Christina Valsamis, Claudia Halaby, Melodi Pirzada

**Affiliations:** Department of Pediatric Pulmonology, Winthrop University Hospital, Mineola, NY, USA

## Abstract

Persistent tachypnea and failure to thrive during infancy have a broad differential diagnosis which includes pulmonary and cardiovascular disorders. Diffuse alveolar hemorrhage (DAH) is a rare entity in children. DAH requires an extensive work-up as certain conditions may need chronic therapy. Cardiovascular disorders are included in the etiology of DAH. We present a case of an 8-month-old female with a moderate, restrictive patent ductus arteriosus (PDA) admitted to the hospital with respiratory distress and failure to thrive. An extensive work-up into tachypnea including multiple echocardiograms did not find an etiology. Open lung biopsy was performed and consistent with pulmonary hypertension. After closure of the PDA, patient's tachypnea improved, and she was discharged home with periodic follow-up showing a growing, thriving child. When an infant presents with tachypnea, a respiratory viral illness is often a common cause. The diagnosis of persistent tachypnea requires further investigation. Echocardiography, although readily available, may not always be sensitive in detecting clinically significant pulmonary hypertension. A clinician must have a heightened index of suspicion to proceed in evaluating for causes of tachypnea with a nonrespiratory etiology.

## 1. Introduction

Tachypnea in infancy has potentially many etiologies. It can be associated with respiratory or nonrespiratory disorders (i.e., cardiac, gastrointestinal, and metabolic). Tachypnea may also be related to a physiologic response to a stimulus such as fever, pain, and/or anxiety. The cause for the tachypnea may be elicited by the history or physical examination. In some patients, a more extensive laboratory and imaging work-up is needed in order to elucidate the cause of tachypnea.

We are presenting a case of an infant with persistent tachypnea since birth. Flexible bronchoscopy with bronchoalveolar lavage (BAL) yielded hemosiderin-laden macrophages which is concerning for alveolar hemorrhage. The cause of alveolar hemorrhage was revealed to be secondary to a restrictive PDA that was initially thought to have minimal hemodynamic and respiratory effects.

## 2. Case Presentation

Eight-month-old female was admitted to the hospital with respiratory distress. She had a history of multiple hospitalizations for viral induced respiratory distress. In the interim period, she was found to be tachypneic at baseline and with failure to thrive. Her medical history was also significant for a right multicystic dysplastic kidney and a moderate restrictive PDA. At the initial presentation of this most recent episode, she was seen by her cardiologist as outpatient and was evaluated for congestive heart failure as a cause of tachypnea. It was recommended to continue furosemide and to be hospitalized due to hypoxemia (O_2_ saturation in mid-80's) and respiratory distress.

This was her fourth hospitalization since birth. The most recent hospitalization in the PICU was for acute respiratory failure requiring mechanical ventilation for one week secondary to human metapneumovirus infection. She was followed by nephrology for right multicystic dysplastic kidney and cardiology for moderate, restrictive PDA and left heart dilation.

On physical exam, she was afebrile, tachypneic (70–90 breaths per minute), with oxygen saturation of 85%. Patient's weight was below the third percentile; she had mild generalized hypotonia, with nasal flaring and subcostal retractions, but she had good air entry bilaterally, and was clear to auscultation.

**Figure 1:**
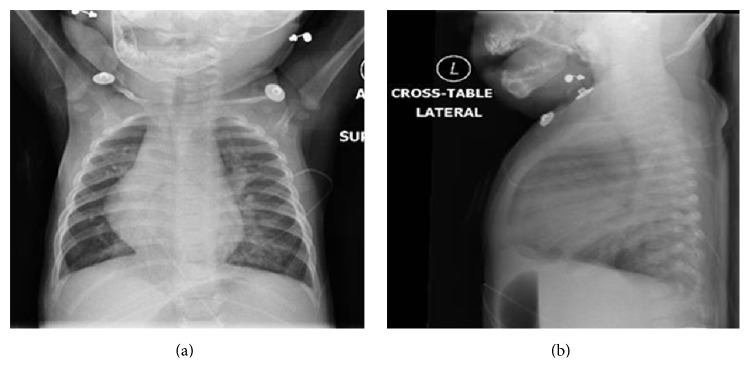
Initial CXR: enlarged cardiac silhouette, with increased interstitial markings (suspected to be from pulmonary edema secondary to left-to-right shunt due to PDA or from a viral process).

Initial evaluation in the emergency room included CBC with differential, basic metabolic panel and blood culture which were within normal. Viral PCR panel was positive for rhinovirus/enterovirus. Chest X-ray (CXR) showed an enlarged cardiac silhouette, with increased interstitial markings which were suspected to be from pulmonary edema secondary to left-to-right shunt due to PDA or from a viral process (Figure 1). She was admitted for further treatment and evaluation of her hypoxia and respiratory distress.

The initial impression was that her four hospitalizations for respiratory distress and tachypnea were secondary to reactive airway disease triggered by viral infections. In addition to reactive airway disease, parents reported coughing and choking with feeds which was concerning for gastroesophageal reflux and dysphagia. She was started on albuterol via nebulizer every 3 hours, a course of systemic corticosteroids, and antireflux medication. While her hypoxemia resolved, her tachypnea persisted.

**Figure 2:**
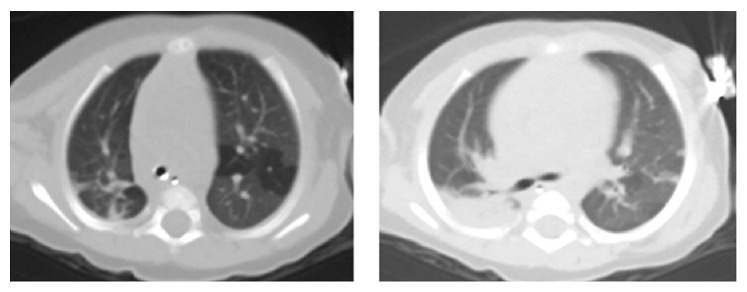
CT chest: bibasilar atelectasis with several areas of air trapping bilaterally. No definite evidence of interstitial lung disease. Enlarged heart.

Feeding/swallow evaluation revealed mild to moderate dysphagia with 90% penetration on video fluoroscopic swallow study. Feeding therapy recommended nectar consistency. Subsequently, her feeds were changed to nasogastric feeding to reduce the risk of aspiration. Despite this management, she remained tachypneic (70–90 breaths per minute). Further evaluation included a normal sweat test and a chest CT which was significant only for bilateral areas of hyperinflation (Figure 2).

Echocardiogram showed moderate tortuous highly restrictive PDA with left-to-right shunt, mild left atrial and left ventricular (LV) enlargement, and a small PFO with left-to-right shunt. Overall LV function was normal without evidence of pulmonary hypertension.

Multiple medical specialties evaluated the patient to determine an etiology for failure to thrive, hypotonia, and persistent tachypnea. Neurology evaluation including head MRI and nerve conduction studies revealed no cause for her hypotonia. Endocrinology evaluation for her failure to thrive (weight less than 3rd percentile and microcephaly since birth) included thyroid studies and basic metabolic work-up which were normal. Genetics evaluation revealed normal karyotype and microarray study.

Repeat echocardiograms showed improving left heart dilation, a restrictive PDA which was smaller (compared to previous studies), and normal ventricular function. Her furosemide was discontinued which did not worsen her respiratory status or her cardiomegaly.

A multidisciplinary meeting was held, due to her persistent tachypneic and poor weight gain. The cardiologist's impression was that the PDA was less likely to be the cause of tachypnea. The consensus agreement was that her tachypnea was likely secondary to a pulmonary etiology.

As a next step in evaluating her tachypnea and failure to thrive, a flexible bronchoscopy with BAL was performed. BAL yielded a bloody fluid; however, no iatrogenic trauma was suspected during the procedure. The BAL fluid was positive for hemosiderin-laden macrophages. Therefore, a work-up for alveolar hemorrhage was pursued. In consultation with the allergy-immunology service for suspected Heiner's syndrome (cow's milk hypersensitivity induced pulmonary disease), she was changed to a hypoallergenic formula; however, there was no improvement in her respiratory status. Milk allergy and precipitins were negative. Rheumatology evaluation for immune-mediated alveolar hemorrhage was unremarkable.

**Figure 3:**
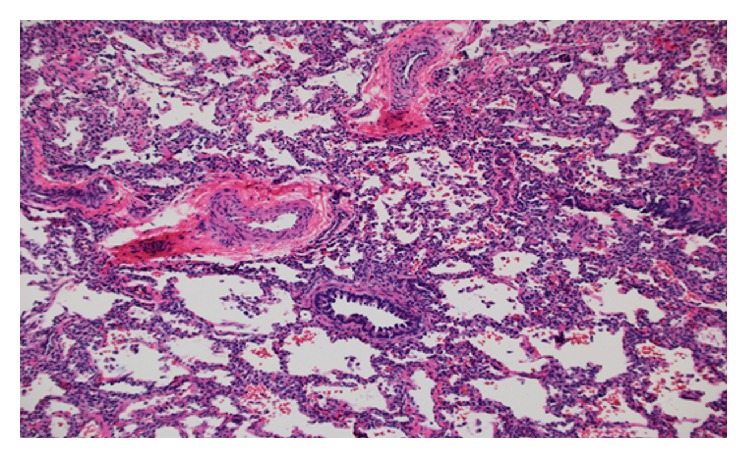
Pulmonary arteriopathy with moderate medial hypertrophy and muscularization of the intralobular arterioles.

After a thorough investigation, it was decided to proceed to lung biopsy to evaluate for interstitial lung disease. Specimens from the lingular lobe showed mild alveolar simplification and pulmonary arteriopathy with moderate hypertrophy and muscularization of the intralobular arterioles which was suggested to be secondary to pulmonary arterial hypertension due to her PDA (Figure 3). The patient was then transferred to a center for cardiac catheterization. Cardiac catheterization was performed and did not reveal pulmonary hypertension (patient's mean pulmonary arterial pressures <25 mmHg). The PDA was coiled during the catheterization.

She was transferred back to our facility. On reevaluation, her respiratory rate was noted to be 40–50 breaths per minute compared to 90 breaths per minute prior to PDA closure. Oral feeds were initiated and the nasogastric tube was removed. The patient started having significant weight gain postclosure of her PDA. She was discharged home 10 days after cardiac catheterization. Subsequent outpatient visits showed consistent age appropriate weight gain and stable respiratory rates.

## 3. Discussion

Persistent tachypnea has a wide differential diagnosis [1]. It can be due to respiratory or nonrespiratory etiologies. When a child presents with tachypnea, the clinician should proceed in a systematic fashion when considering a varied differential diagnosis.

After analysis of the BAL which was positive for hemosiderin-laden macrophages, an evaluation for alveolar hemorrhage ensued. Pulmonary hemorrhage may be alveolar (diffuse) or focal [2]. Pulmonary hemorrhage can arise from either the bronchial or pulmonary circulation.

The bronchial circulation is a high-pressure, low-volume circuit supplied by the bronchial arteries. Origin of the bronchial arteries most often arise directly from the aorta or one of its branches. These vessels provide blood to the conducting airways. Since the bronchial circulation is a high-pressure system, bleeding has the potential to be profuse, sometimes resulting in exsanguination [3–5]. In contrast, the pulmonary circulation is a low-pressure, high-volume circuit that arises from the right ventricle and provides blood flow to the gas exchange units of the respiratory airways. Disruption of this system results in alveolar hemorrhage, which is often low-grade, chronic, and more diffuse [4].

Diffuse alveolar hemorrhage (DAH) is a potentially life-threatening condition but fortunately a rare entity in children. There is a spectrum of disorders associated with DAH, but due to its rare entity no prospective studies have been done to estimate its relative frequency or prevalence [6]. DAH arises from the small vessels and capillaries of the pulmonary circulation. Presence of hemosiderin-laden macrophages is a key sign for DAH. Macrophages are responsible for clearing debris (i.e., free erythrocytes) from the lung. In one study looking at hemosiderin-laden macrophages, they first appeared 3 days following a hemorrhagic episode, peaked at days 7 to 10, and were still found at 2 months in 10% of macrophages [7]. Although hemosiderin-laden macrophages are sensitive for pulmonary bleeding, it is not specific for the cause of the bleeding [7].

DAH can present as a spectrum from severe (i.e., life-threatening respiratory failure) to a more subtle presentation with mild or minimal symptoms such as cough or as in our patient tachypnea. Crackles, rhonchi, and/or wheezes may be present on auscultation but are generally nonspecific. A lack of prominent respiratory symptoms does not exclude a diagnosis of DAH [8]. Some patients may present with pallor and fatigue related to chronic anemia and poor weight gain or failure to thrive.

DAH is diverse and includes pulmonary capillaritis, drugs, toxins, allogeneic hematopoietic stem cell transplantation (HCT), coagulopathies, immune-mediated cause, and blast lung injury [6]. Cardiovascular disorders must be considered in children who present with persistent tachypnea and DAH [8]. These include entities such as mitral stenosis, pulmonary venoocclusive disease, pulmonary capillary hemangiomatosis, and pulmonary telangiectasia, which can result in a congestive vasculopathy with resultant hemosiderin-laden macrophages from DAH [8]. Pulmonary hypertension and pulmonary embolism can both cause hemosiderin-laden macrophages and should be in the differential diagnosis of DAH [9]. Suspicion for left-sided heart lesions, such as mitral stenosis or pulmonary venoocclusive disease, warrants a cardiac evaluation, including electrocardiogram and echocardiogram. Cardiac catheterization may be indicated to make a definitive diagnosis.

Our patient had multiple echocardiograms that did not reveal findings consistent with pulmonary hypertension. The PDA was thought to be hemodynamically insignificant due to its restrictive nature. Some authors recommend lung biopsy to be performed in any child presenting with DAH of unclear cause, particularly after cardiovascular causes have been ruled out [7]. The lung biopsy in our patient showed evidence of pulmonary hypertension. However, subsequent cardiac catheterization did not show elevated pulmonary pressures. Therefore, this presents an important clinical question; do persistent PDA's outside the neonatal period have histopathologic changes consistent with pulmonary hypertension and may these be clinically significant despite normal echocardiograms?

Healthy infants with small, restrictive, and hemodynamic insignificant PDAs are not an indication for closure. In our infant with multiple coexisting medical conditions, closure was indicated and was helpful. Cardiac catheterization may not always show elevated pulmonary arterial pressures. In certain patients (i.e., congenital heart disease), pulmonary hypertensive vascular disease (PHVD) may not be present, especially if intracardiac shunts with increased pulmonary blood flow exist [10]. Our patient's multiple coexisting medical conditions made diagnosis difficult, because it did not fit the criteria for pulmonary hypertension. Lung biopsy diagnosed pulmonary hypertension in our patient. Histopathological features of pulmonary hypertension are intimal thickening and medial hypertrophy of the small muscular pulmonary arteries were present [10].

PDA's associated with DAH have been reported, but in preterm infants [11, 12]. The pathophysiologic features of a PDA depend on the extent of the left-to-right shunt and on the cardiac and pulmonary responses to the shunt. The increase in left ventricular pressure increases pulmonary venous pressure, which contributes to pulmonary congestion. As in preterm infants, the overcirculation of the pulmonary blood flow produces an increase in pulmonary arterial pressure which shifts downstream towards the capillary fluid filtration sites. This shift downstream can increases the rate of fluid excretion into the pulmonary interstitium.

In our patient, we suspected a process of disruption of the alveolar capillary interface from increased pulmonary arterial pressures due to the PDA coupled with frequent viral infections, allowing red blood cells to flood the alveolar space, leading to DAH. This can worsen the left-to-right shunt and alter the distribution of pulmonary pressures downstream causing pulmonary edema and pulmonary hemorrhage [13–15].

## 4. Conclusion

We present a case of an infant with persistent tachypnea and an evaluation that led us to the diagnosis of alveolar hemorrhage and pulmonary hypertension from a PDA. Persistent tachypnea in infancy has a myriad of causes. It can be due to respiratory or nonrespiratory disease.

Investigation into the etiology of tachypnea may require an extensive work-up with laboratory, imaging, and/or invasive procedures. The restrictive PDA, although it is thought to be hemodynamically stable, was the cause of her tachypnea secondary to pulmonary overcirculation. This case brings to the surface that, despite being thought of as hemodynamically insignificant, PDAs in children with coexisting medical conditions can be a cause for tachypnea, diffuse alveolar hemorrhage, and pulmonary hypertension.

## Abbreviations

DAH: Diffuse alveolar hemorrhage
PDA: Restrictive patent ductus arteriosus
BAL: Bronchoalveolar lavage
CXR: Chest X-ray
LV: Left ventricle.
